# Efficacy of a Low Dose of Estrogen on Antioxidant Defenses and Heart Rate Variability

**DOI:** 10.1155/2014/218749

**Published:** 2014-03-10

**Authors:** Cristina Campos, Karina Rabello Casali, Dhãniel Baraldi, Adriana Conzatti, Alex Sander da Rosa Araújo, Neelam Khaper, Susana Llesuy, Katya Rigatto, Adriane Belló-Klein

**Affiliations:** ^1^Universidade Federal do Rio Grande do Sul, Sarmento Leite, 500 Bairro Farroupilha, 90050-170 Porto Alegre, RS, Brazil; ^2^Instituto de Cardiologia do Rio Grande do Sul, 90620-001 Porto Alegre, RS, Brazil; ^3^Medical Sciences Division, Northern Ontario School of Medicine, Lakehead University, Thunder Bay, ON, Canada P7B 5E1; ^4^Universidad de Buenos Aires, C1053ABJ Buenos Aires, Argentina; ^5^Universidade Federal de Ciências da Saúde de Porto Alegre, 90050-170 Porto Alegre, RS, Brazil

## Abstract

This study tested whether a low dose (40% less than the pharmacological dose of 17-**β** estradiol) would be as effective as the pharmacological dose to improve cardiovascular parameters and decrease cardiac oxidative stress. Female Wistar rats (*n* = 9/group) were divided in three groups: (1) ovariectomized (Ovx), (2) ovariectomized animals treated for 21 days with low dose (LE; 0.2 mg), and (3) high dose (HE; 0.5 mg) 17-**β** estradiol subcutaneously. Hemodynamic assessment and spectral analysis for evaluation of autonomic nervous system regulation were performed. Myocardial superoxide dismutase (SOD) and catalase (CAT) activities, redox ratio (GSH/GSSG), total radical-trapping antioxidant potential (TRAP), hydrogen peroxide, and superoxide anion concentrations were measured. HE and LE groups exhibited an improvement in hemodynamic function and heart rate variability. These changes were associated with an increase in the TRAP, GSH/GSSG, SOD, and CAT. A decrease in hydrogen peroxide and superoxide anion was also observed in the treated estrogen groups as compared to the Ovx group. Our results indicate that a low dose of estrogen is just as effective as a high dose into promoting cardiovascular function and reducing oxidative stress, thereby supporting the approach of using low dose of estrogen in clinical settings to minimize the risks associated with estrogen therapy.

## 1. Introduction

The risk of cardiovascular disease (CVD) increases dramatically in the postmenopausal women as compared to the premenopausal women. Estrogen helps to protect women against CVD during the childbearing years and, after menopause, the CVD can be prevented or at least reduced by estrogen therapy [1–3]. It has been demonstrated that estrogen therapy can reduce many risk factors, improving lipid profile and glucose metabolism [1].

The increased risk of CVD in menopause is also accompanied by oxidative stress, a condition when there is an increase in reactive oxygen species (ROS) levels which may cause oxidative damage to cells [4]. On the other hand, cells have mechanisms to protect from ROS mediated toxicity. Glutathione (GSH) is the major nonenzymatic antioxidant and participates in many cellular reactions of ROS scavenging. In such reactions, GSH is oxidized to form glutathione disulfide (GSSG). An increase in the redox ratio which is represented by GSH/GSSG is indicative of reduced oxidative stress [5]. An impairment in redox balance plays an important role in the reduced nitric oxide bioavailability which may ultimately affect the sympathovagal balance (SVB) [6, 7]. Moreover, some studies have reported a link between menopause and SVB impairment [8, 9] suggesting a role of estrogen in the autonomic nervous control of the cardiovascular system.

Power spectral analysis of heart rate variability (HRV) is a noninvasive method to assess SVB [10]. Alterations in HRV, which primarily reflect the tonic autonomic modulation, may have substantial clinical implications. Low HRV, which has been shown in postmenopausal women, is associated with an increased risk of CVD [11]. In addition, some studies indicate that menopausal women have a sympathovagal imbalance and that estrogen improves the SVB centrally and peripherally by decreasing sympathetic and increasing parasympathetic tone [12].

Estrogen therapy improves women's quality of life [13] and is widely used for controlling typical menopausal symptoms such as vaginal atrophy, hot flushes, osteoporosis, and sleep disturbances [14]. However, at standard pharmacological doses, several adverse effects, including higher risk of breast cancer, stroke, and venous thromboembolism, outweigh the benefits of estrogen therapy [15].

As cardiovascular diseases are highly prevalent after menopause [16] and estrogen is the most commonly used treatment to reduce menopause symptoms [13], the need to find a safer estrogen dose to control menopause related discomforts has been recommended. Indeed, studies evaluating different regimens of hormone therapy have demonstrated that a low dose of estrogen is associated with a significant decrease in mammographic density [17]. According to Mercuro et al. [18], low doses of estrogen are just as effective as conventional doses to improve the lipid profile and the endothelial function. Moreover, a low dose of estrogen has demonstrated to be effective for the alleviation of climacteric symptoms [19] and it has good tolerability associated with a low incidence of the most common side effects [20].

There have been no studies to date that have tested the effects of low dose of estrogen on oxidative stress and its association with the cardiac autonomic control in ovariectomized rats. Thus, the aim of this study was to test whether the treatment with a low dose of 17-*β* estradiol to ovariectomized rats could be as effective as a pharmacological dose to reduce the cardiac oxidative stress and improve the SVB.

## 2. Methods

### 2.1. Drugs and Reagents

Ketamine hydrochloride was purchased from König Lab S.A., SP, Brazil, and xylazine, from Virbac do Brazil I.P., SP, Brazil. 17-*β* estradiol and all other drugs/reagents were purchased from Sigma Chemical Co., St. Louis.

### 2.2. Animals and Groups

In total, 27 female Wistar rats (body weight 200–230 g) from the animal care of the Federal University of Rio Grande do Sul, Brazil, were kept at 20–22°C in a 12 : 12 h dark/light cycle. They were subjected to bilateral ovariectomy under ketamine hydrochloride (80 mg/kg i.p.) and xylazine (16 mg/kg i.p.) anesthesia. After one week following ovariectomy, each ovariectomized animal received subcutaneously (under ketamine and xylazine anesthesia) silastic capsules either filled with 17-*β* estradiol diluted in sunflower oil (treated groups) or only sunflower oil as a vehicle (ovariectomized control group). Rats were divided into three experimental groups (*n* = 9, per group): (1) ovariectomized (Ovx) receiving only sunflower oil, (2) animals treated with 40% of the pharmacological (LE; 0.2 mg/pellet for 21 days) dose of estradiol, and (3) animals treated with a pharmacological (HE; 0.5 mg/pellet for 21 days) dose of estradiol [21]. All animals had access to water and regular rodent chow ad libitum. All procedures were approved by the Institutional Animal Care Ethics Committee and the experiments were conducted in accordance with the Guide for the Care and Use of Laboratory Animals (US Department of Health and Human Services, NIH publication number 86-23).

### 2.3. Hemodynamic Measurements

Under anesthesia (ketamine 80 mg/kg, i.p.; xylazine 16 mg/kg, i.p.), the left carotid artery was cannulated with a PE 50 catheter connected to a strain gauge transducer (Narco Biosystem Pulse Transducer RP-155, Houston, TX, USA) linked to a pressure amplifier (HP 8805C, Hewlett Packard). Pressure readings were recorded on a microcomputer equipped with an analog-to-digital conversion board (WinDaq, 2 kHz sampling frequency; DataQ Instruments, Inc., Akron, OH). The catheter was advanced into the left ventricle (LV) to record the left ventricular systolic pressure (LVSP, mmHg), the left ventricular end-diastolic pressure (LVEDP, mmHg), +*dP/dt* (mmHg/s), −*dP/dt* (mmHg/s), and heart rate (HR). After hemodynamic measurements, animals were sacrificed by decapitation for heart and blood collection.

### 2.4. Autonomic Evaluation

After detecting the pulse intervals, the heart rate was automatically calculated on a beat-to-beat basis as the time interval between two consecutive systolic peaks or pulse interval (PI). All detection was carefully checked to avoid erroneous or missed beats. Sequences of 150–160 beats were randomly chosen and if there was an inconsistent pattern, it was discarded and a new random selection was performed. Frequency domain analysis of HRV was performed with an autoregressive algorithm [22] on the PI interval sequences (tachograms) and on respective systolic sequences (cystograms). The power spectral density was calculated for each time series. In this study, two spectral components were considered: low frequency (LF), from 0.10 to 1.00 Hz and high frequency (HF), from 1.00 to 5.00 Hz. The spectral components were expressed in absolute (abs) and normalized units (nu). Normalization consisted of dividing the power of a given spectral component by the total power, then multiplying the ratio by 100 [23]. All recordings were performed in a sound attenuated room. The ratio of LF/HF, as an index of SVB, was also calculated.

### 2.5. Plasma Hormone Concentration

Plasma estradiol was measured by electrochemiluminescence (Roche Diagnostics) at the Weinmann Clinical Analysis Laboratory. Briefly, this test employs the principle of competitive assay using a polyclonal antibody against the 17-*β* estradiol.

### 2.6. Hydrogen Peroxide Concentration

The assay was based on the horseradish peroxidase- (HRPO-) mediated oxidation of phenol red by H_2_O_2_, leading to the formation of a compound measureable at 610 nm. Heart slices were incubated for 30 min at 37°C in 10 mmol/L phosphate buffer consisting of 140 mmol/L NaCl and 5 mmol/L dextrose. The supernatants were transferred to tubes with 0.28 mmol/L phenol red and 8.5 U/mL HRPO. After 5 min incubation, 1 mol/L NaOH was added and it was read at 610 nm. The results were expressed in nmol H_2_O_2_/g tissue [24].

### 2.7. Determination of Superoxide Anion Concentration

Superoxide anion concentration was determined in heart mitochondrial samples isolated by centrifugations. It was based on the spectrophotometric measurement of the epinephrine oxidation reaction in which superoxide anion is a reactant, leading to the formation of a compound measureable at 480 nm. The results were expressed in mmol/mg protein [25].

### 2.8. Preparation of Heart Homogenates for Analysis of Antioxidants

Hearts were homogenized in an ultra-Turrax blender using 1 g of tissue for 5 mL of 150 mmol/L potassium chloride added to 20 mmol/L phosphate buffer, pH 7.4. The homogenates were centrifuged at 1000 g for 20 min at −2°C as described elsewhere [26].

### 2.9. TRAP

Total antioxidant capacity (TRAP) was measured by chemiluminescence using 2,2′-azo-bis(2-amidinopropane) (ABAP, a source of alkyl peroxyl free radicals) and luminol. A mixture consisting of 20 mmol/L ABAP, 40 *μ*mol/L luminol, and 50 mmol/L phosphate buffer (pH 7.4) was incubated to achieve a steady-state luminescence from the free radical-mediated luminol oxidation. A calibration curve was obtained by using different concentrations (between 0.2 and 1 *μ*mol/L) of Trolox (hydrosoluble form of vitamin E). Luminescence was measured in a liquid scintillation counter using the out-of-coincidence mode and the results were expressed in units of Trolox/mg protein [27].

### 2.10. Determination of Total and Oxidized Glutathione Concentration

To determine oxidized (GSSG) and total glutathione concentration, tissue was homogenized in 2 mol/L perchloric acid and centrifuged at 1000 g for 10 min and 2 mol/L potassium hydroxide was added to the supernatant. The reaction medium contained 100 mmol/L phosphate buffer (pH 7.2), 2 mmol/L NADPH, 0.2 U/mL glutathione reductase, and 70 *μ*mol/L 5,5′ dithiobis (2-nitrobenzoic acid). To determine oxidized glutathione, the supernatant was neutralized with 2 mol/L potassium hydroxide and inhibited by the addition of 5 *μ*mol/L N-ethylmaleimide and absorbance was read at 420 nm [28]. Reduced glutathione (GSH) values were determined from the total and GSSG concentration. The redox status was represented by the GSH/GSSG ratio.

### 2.11. Determination of Antioxidant Enzyme Activities

Superoxide dismutase activity was expressed as units per milligram of protein and is based on the inhibition of superoxide radical reaction with pyrogallol [29]. Catalase activity was determined in heart homogenates by following the decrease in absorption of hydrogen peroxide. It was expressed as pmol/mg protein [30]. Protein was measured in heart homogenates, using bovine serum albumin as described by Lowry et al. [31].

### 2.12. Statistical Analysis

Data are shown as mean ± standard deviation. Statistical analyses were performed using one-way ANOVA followed by Student Newman-Keuls post hoc test. The Pearson correlation was used to assess the association among variables. *P* < 0.05 was considered significant.

## 3. Results

### 3.1. Ovariectomy and Estradiol Therapy

As expected, the ovariectomy decreased plasma estrogen concentration and 17-*β* estradiol treatment increased its concentration (LE = 587 ± 19 pg/L, HE = 1813 ± 37 pg/L versus Ovx = 58 ± 6 pg/L). This result is in consonance with Paigel et al. [32], who observed serum estrogen levels in ovariectomized rats similar to those observed by us. Moreover, in ovary-intact animals, Paigel et al. [32] found estrogen serum concentration of about 120 pg/L. The 17-*β* estradiol treatment also significantly (*P* < 0.001) decreased the body weight and increased the uterine weight (Table 1), confirming the effectiveness of hormonal treatment.

### 3.2. Hemodynamic Parameters

The LVEDP, which is a diastolic function, was significantly (*P* < 0.05) decreased in LE (by 60%) and HE (by 35%) groups when compared to Ovx animals. Moreover, no changes were found in +*dP/dt*, a cardiac contractility index, and −*dP/dt*, a cardiac relaxation index, LVSP, and HR, among any of the groups (Table 2).

### 3.3. Autonomic Evaluations

Hfabs, which represents the parasympathetic drive, and HRV were significantly higher (*P* < 0.05) in both the HE and LE groups as compared to the Ovx group. LFabs, LFnu, HFnu, and LF/HF ratio did not show any statistically significant differences with estrogen treatment (Table 3).

### 3.4. Reactive Oxygen Species Concentrations

**Figure 1:**
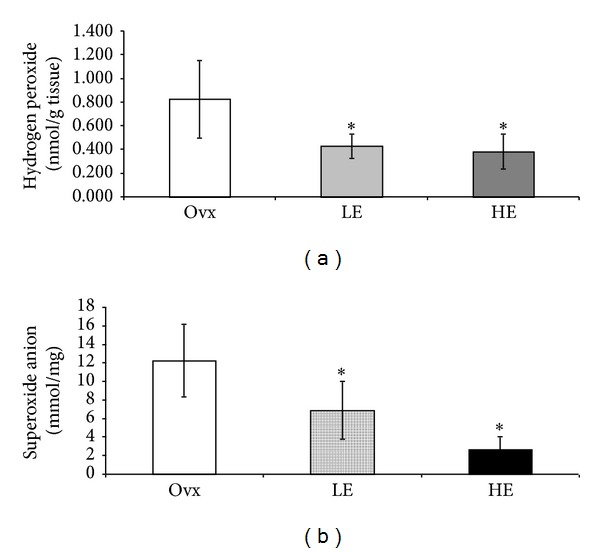
(a) Hydrogen peroxide concentration in myocardium (in nmol/g tissue) and (b) superoxide anion concentration in myocardium (in mmol/mg protein). Data are mean ± SD. *N* = 9 per group. Ovariectomized group = Ovx; ovariectomized group treated for 21 days with low dose of estrogen = LE; ovariectomized group treated for 21 days with high dose of estrogen = HE. *P < 0.05 versus Ovx.

Cardiac H_2_O_2_ concentration (in nmol/g tissue) was significantly decreased (*P* < 0.05) in estrogen groups as compared to the Ovx group (LE = 0.43 ± 0.10; HE = 0.38 ± 0.14 versus Ovx = 0.83 ± 0.32 (Figure 1(a)). Similarly, the cardiac superoxide anion concentration (mmol/mg protein) was significantly (*P* < 0.05) decreased in the treated groups (LE = 6.87 ± 3.13; HE = 2.65 ± 1.37) as compared to the Ovx group (Ovx = 12.22 ± 3.90) (Figure 1(b)).

### 3.5. TRAP, Glutathione Concentration, and the Redox Ratio

Total antioxidant capacity was significantly higher in estrogen treated groups as compared to the Ovx group. Moreover, a strong positive correlation between TRAP and HRV (*r* = 0.8922; *P* < 0.01) was also observed. GSSG levels decreased in the HE group as compared to Ovx group. The redox (GSH/GSSG) ratio, which is an index of oxidative stress, and GSH were significantly (*P* < 0.05) higher in both the estrogen treated groups when compared to the Ovx group (Table 4).

### 3.6. Antioxidant Enzyme Activities

**Figure 2:**
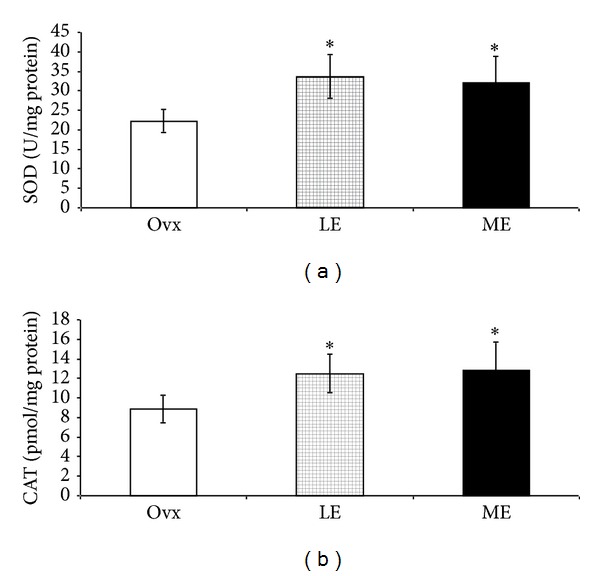
(a) Superoxide dismutase activity (U/mg protein) and (b) catalase activity (pmol/mg protein). Data are mean ± SD. *N* = 9 per group. Ovariectomized group = Ovx; ovariectomized group treated for 21 days with low dose of estrogen = LE; ovariectomized group treated for 21 days with high dose of estrogen = HE. *P < 0.05 versus Ovx.

SOD and CAT activities were significantly higher in estrogen treated groups (SOD in U/mg protein: LE = 33.65 ± 5.54; HE = 32.10 ± 6.80; CAT in pmol/mg protein: 12.5 ± 2.0; 12.8 ± 2.9) as compared to the Ovx group (SOD: 22.24 ± 3.00; CAT: 8.9 ± 1.4), and there was no difference between HE and LE groups (Figures 2(a) and 2(b)).

## 4. Discussion

The present study showed for the first time that a low dose of estrogen is just as effective as a high dose to improve the antioxidant reserve and reduces cardiac oxidative stress. This was associated with a lower LVEDP and higher HRV, which signifies reduced cardiovascular risk.

In this study, we reported a significant decrease in LVEDP in both estrogen-treated groups. This result is in agreement with a study from Bhuiyan et al. [33] who demonstrated similar values of LVEDP in ovariectomized rats. Moreover, we did not observe significant differences in +*dP/dt*, an index of myocardial contractility, or −*dP/dt*, an index of myocardial relaxation, neither in LVSP and HR. Furthermore, our study also is in consonance with Nekooeian and Pang [34] who documented a decrease in LVEDP in rats treated with a pharmacological dose of estrogen. This result suggests a reduction in afterload with maintained systolic function. Indeed, in another study we observed that estrogen therapy induces an increase in aortic nitric oxide bioavailability, resulting in an increase in vasodilation and blood pressure reduction [7]. Estradiol has been reported to play a role in mediating a reduction in blood pressure in hypertensive female animal models [7, 35, 36]. Moreover, since nitric oxide is reported to increase diastolic distensibility [37], estrogen treatment could prevent the increase of LVEDP by increasing nitric oxide synthase activity in the heart as reported by others [38].

We did not find changes in LFa, LFnu, HFnu, and LF/HF ratio in the two treated groups. These results are consistent with Schuchert et al. [39] who have also demonstrated no changes in these parameters after estrogen treatment. On the other hand, HFabs, an important index of cardiac parasympathetic modulation [23], was significantly improved after estrogen treatment representing reduced cardiovascular risk [40]. HRV was also increased in the estrogen treated groups. This result is in agreement with Liu et al. [41] who have reported that estrogen therapy is able to improve cardiac autonomic control. Although no changes were found in HR, our results demonstrated that there was a significant increase in HRV after estrogen treatment. This result highlights the effectiveness of estrogen, even in a low dose, to increase HRV and potentially lower the risk factors for cardiovascular complications [42]. More importantly, in our current study, we also found a positive correlation between HRV and TRAP (*r* = 0.8922; *P* < 0.01), suggesting that an increase in the antioxidant capacity might contribute to the improvement in cardiac autonomic control. This association supports the hypothesis that estrogen administration increases nonenzymatic antioxidants, which improves cardiac autonomic control and reduces oxidative stress. According to Semen et al. [43], a decrease in oxidative stress results in an improved HRV. It has also been reported that estrogen therapy leads to an increase in total serum antioxidant capacity resulting in an improvement in the antioxidant status in women [44]. Accordingly, in the treated groups, we have observed an increase in TRAP that represents an index of nonenzymatic antioxidants, especially the hydrosoluble ones. One possible explanation to this preservation in the nonenzymatic antioxidants could be the enhanced antioxidant enzyme activity. In fact, SOD and CAT activities were significantly higher after estrogen treatment. These results are in agreement with others who have reported that estradiol has antioxidant properties whereby it increases CAT [45] and SOD activities and decreases NADPH oxidase enzyme activity and superoxide production [7, 45–47]. In the present study a significant decrease in cardiac concentrations of superoxide anion and hydrogen peroxide in animals treated with estrogen was also documented. These results are in consonance with a study of Lam et al. [48] who demonstrated a significant decrease in superoxide anion production in aortas from ovariectomized rats treated with estrogen. According to our results, the low dose was also able to decrease these ROS concentrations. Additionally, it is widely recognized that estrogen exhibits protective antioxidant effects through the phenolic hydroxyl group of 17-*β* estradiol that can act as a ROS scavenger [49]. Our findings do suggest that estrogen, even in a low dose, is able to improve the antioxidant defenses and decrease ROS concentrations.

Indeed, GSH/GSSG ratio was significantly increased and GSSG was decreased in our treated groups. These results indicate that there was a reduction in oxidative stress after estrogen treatment. Our data are in agreement with Baeza et al. [50], who also demonstrated that estrogen in a conventional dose was able to decrease oxidative stress in liver, heart, and kidney from ovariectomized rats.

This scenario, where antioxidants are increased and ROS concentration is decreased, contributes to a more favorable redox balance.

## 5. Conclusion

In conclusion, based on our results, estrogen therapy, even in a low dose, reduced cardiac ROS concentration and increased enzymatic and nonenzymatic antioxidants in ovariectomized rats. This was reflected in improved left ventricle function and cardiac autonomic control. Once these cardioprotective effects were similar in low and high dose of estrogen, it is reasonable to recommend low doses in clinical settings to avoid undesirable side effects associated with the high dose.

## Figures and Tables

**Table 1:** Morphometrics data.

	Ovx (*N* = 9)	LE (*N* = 9)	HE (*N* = 9)
Uterine weight (g)	0.15 ± 0.01	0.63 ± 0.02*	0.89 ± 0.13^∗†^
Body weight (g)	234 ± 11	209 ± 9*	210 ± 7*

Data are mean ± SD. Ovx: ovariectomized group; HE: high dose estrogen-treated group; LE: low dose estrogen-treated group. *P < 0.05 versus Ovx; ^†^ *P* < 0.05 versus LE.

**Table 2:** Left ventricular hemodynamic parameters.

	Ovx (*N* = 5)	LE (*N* = 5)	HE (*N* = 5)
LVEDP (mmHg)	12.17 ± 4.54	5.10 ± 1.98*	7.95 ± 2.21*
LVSP (mmHg)	101.71 ± 12.78	120.91 ± 20.71	110.40 ± 7.51
HR (bpm)	212 ± 12.78	221 ± 22.59	183.81 ± 20.83
+dP/dt (mmHg/s)	5809 ± 924	6435 ± 549	5505 ± 450
−dP/dt (mmHg/s)	−3946 ± 786	−5262 ± 890	−4098 ± 251

Data are mean ± SD. Ovx: ovariectomized group; HE: high dose estrogen-treated group; LE: low dose estrogen-treated group. *P < 0.05 versus Ovx.

**Table 3:** Power spectral analysis.

	Ovx (*N* = 5)	LE (*N* = 5)	HE (*N* = 5)
HRV (ms^2^)	14.34 ± 3.56	39.98 ± 11.00*	69.62 ± 27.32*
LFabs (ms^2^)	2.49 ± 1.53	5.03 ± 1.89	8.09 ± 7.04
HFabs (ms^2^)	9.78 ± 2.84	28.04 ± 9.718*	53.35 ± 29.96*
LFnu	20.22 ± 5.20	16.67 ± 5.12	11.05 ± 3.03
HFnu	79.77 ± 5.14	83.32 ± 5.10	88.95 ± 15.74
LF/HF	0.25 ± 0.07	0.2 ± 0.07	0.12 ± 0.08

Data are mean ± SD. Ovx: ovariectomized group; HE: high dose estrogen-treated group; LE: low dose estrogen-treated group; HRV: heart rate variability; LFabs: absolute low frequency; HFabs: absolute high frequency; LFnu: normalized low frequency; HFnu: normalized high frequency. *P < 0.05 versus Ovx.

**Table 4:** Myocardial nonenzymatic antioxidant defenses.

	Ovx (*N* = 9)	LE (*N* = 9)	HE (*N* = 9)
TRAP (units of Trolox/mg protein)	25.50 ± 7.96	57.60 ± 24.13*	53.55 ± 16.15*
Total GSH (nmol/mg protein)	0.18 ± 0.08	0.32 ± 0.06*	0.28 ± 0.14*
GSSG (nmol/mg protein)	0.026 ± 0.008	0.022 ± 0.005	0.014 ± 0.005*
GSH/GSSG	5.98 ± 2.45	13.57 ± 2.23*	19.55 ± 9.03*

Data are mean ± SD. Ovx: ovariectomized group; HE: high dose estrogen-treated group; LE: low dose estrogen-treated group. *P < 0.05 versus Ovx.
